# MetaCCI: meta cell–cell interaction inference and its application to CCIs characteristics of MDS

**DOI:** 10.1093/bioinformatics/btag313

**Published:** 2026-05-18

**Authors:** Heewon Park, Seiya Imoto, Satoru Miyano

**Affiliations:** School of Mathematics, Statistics and Data Science, Sungshin Women’s University, Seoul, 02844, Korea; M&D Data Science Center, Institute of Science Tokyo, Tokyo, 113-8510, Japan; Human Genome Center, Institute of Medical Science, University of Tokyo, Tokyo, 108-8639, Japan; Human Genome Center, Institute of Medical Science, University of Tokyo, Tokyo, 108-8639, Japan; M&D Data Science Center, Institute of Science Tokyo, Tokyo, 113-8510, Japan; Human Genome Center, Institute of Medical Science, University of Tokyo, Tokyo, 108-8639, Japan

## Abstract

**Motivation:**

Cell–cell interactions (CCIs) are fundamental to multicellular organisms and play crucial roles in diverse biological processes and disease mechanisms. Understanding CCIs is vital for deciphering disease pathogenesis and developing therapeutic strategies. Although numerous computational methods have been developed to infer CCIs from complex biological data, most existing approaches rely primarily on single-gene expression levels and ligand-receptor databases, often failing to capture the nuanced network-wide changes characteristic of disease states.

**Result:**

We propose MetaCCI, a novel computational strategy that integrates meta-information into CCI inference by extending the traditional gene expression-based analysis to a gene regulatory network framework. MetaCCI meticulously combines established ligand-receptor pairs with quantitative insights into gene behavior within complex gene networks, enabling the precise extraction of relevant targets for CCI inference. Subsequently, CCI inference was performed using an eigen cell co-expression network, providing a more holistic view of cell–cell communication. Monte Carlo simulations demonstrated that MetaCCI consistently outperforms existing methods in CCI inference. We applied MetaCCI to characterize cell–cell communication in Myelodysplastic Syndromes (MDS). Our results identified distinct interaction patterns in MDS compared with normal cell populations, specifically highlighting the loss of CCIs between “Dendritic cells and Hematopoietic precursor cells” and between “Dendritic cells and Hematopoietic multipotent progenitor cells” as characteristic features of MDS. Furthermore, FABP5, CD63, and HMGB1 were identified as MDS-specific markers. These findings suggest that diminished CCIs involving dendritic cells, hematopoietic precursor cells, and multipotent progenitor cells are pivotal to MDS pathogenesis.

**Availability and implementation:**

The MetaCCI software is freely available at https://github.com/HeewonGitHub/MetaCCI. An archived version of the software and example datasets used in this study is available at Zenodo: https://doi.org/10.5281/zenodo.20101527.

## Graphical abstract

**Figure:**
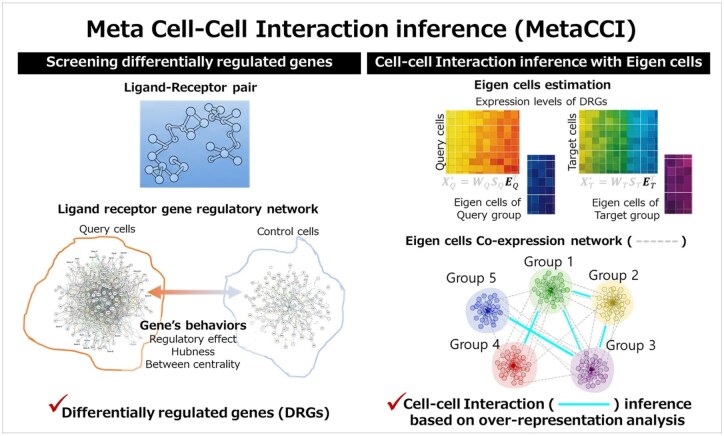

## Introduction

Cell–cell interactions (CCIs) play a fundamental role in complex biological processes in multicellular organisms. They are crucial for the progression and regulation of various diseases and are essential for elucidating disease-related cellular mechanisms. Therefore, a comprehensive understanding of CCIs is vital for deciphering disease pathogenesis and developing novel therapeutic strategies.

To infer CCIs from complex biological data, various computational methods have been proposed. SingleCellSignalR uses a product-based co-expression framework with a regularized ligand–receptor score to account for variable sequencing depth in scRNA-seq data (Cabello-Aguilar *et al.* 2020). CellChat infers CCIs by modeling communication probabilities based on ligand and receptor expression in sender and receiver cell groups, using geometric means for multigene complexes (Jin *et al.* 2025). REMI estimates interaction likelihoods through Pearson correlation of ligand and receptor expression levels (Yu *et al.* 2022). iTALK identifies CCIs by detecting differentially expressed ligands and receptors using differential expression analysis in single-cell data (Tyler *et al.* 2019). Although numerous approaches have been developed to study CCIs in complex diseases, most previous studies focus on single genes and their expression levels. However, disease mechanisms typically arise from dysregulation of complex molecular networks rather than from alterations in individual genes, thus expression-level analyses are insufficient to capture network-wide perturbations. Furthermore, existing methods heavily depend on predefined ligand–receptor databases. Consequently, these approaches often fail to adequately reveal the systemic disruptions that underlie disease-associated cellular mechanisms. Recent studies have begun to incorporate gene regulatory network information into cell–cell communication (CCI) analysis. For example, NicheNet integrates prior knowledge of ligand–target regulatory networks to predict downstream gene expression changes in receiver cells (Browaeys *et al.* 2020). Similarly, the recently proposed strategy LIANA+ extends ligand–receptor analysis by integrating multiple CCI inference methods and incorporating gene regulatory network information into a consensus communication model (Dimitrov *et al.* 2024). While these approaches successfully incorporate regulatory information into CCI analysis, they primarily rely on predefined signaling or gene regulatory networks and are largely designed to predict ligand-driven downstream gene activity. However, these approaches may still have limited ability to capture context-specific regulatory rewiring across cell populations because they depend largely on prior knowledge networks rather than reconstructing regulatory relationships directly from data.

To overcome these limitations, we propose a novel computational framework, MetaCCI, which incorporates metadata into the CCI inference process. MetaCCI extends conventional gene expression–based approaches to a gene network framework, acknowledging that disease mechanisms arise from the collective and interconnected behavior of genes rather than from individual gene expression alone. MetaCCI integrates curated ligand–receptor pairs as foundational communication links with quantitative information describing gene behavior within complex gene networks. By integrating the multifaceted aspects of gene behavior, MetaCCI precisely extracts the most relevant subjects for CCI inference. Subsequently, CCIs are inferred using an eigen cell co-expression network following the DD-CC-II framework (Park *et al.* 2025), enabling the identification of functional modules within cell populations and the inference of interactions between these modules. This integrated strategy provides a more holistic view of cell–cell communication and facilitates biologically interpretable CCI inference by capturing the dynamic, network-driven mechanisms underlying complex diseases. In contrast to existing gene regulatory network (GRN)-integrated CCI frameworks such as NicheNet and LIANA+, MetaCCI adopts a fundamentally different strategy. MetaCCI reconstructs condition-specific GRNs directly from gene expression data. Rather than relying solely on prior regulatory knowledge, MetaCCI employs a data-driven framework to infer GRNs and quantifies gene regulatory activity using network topology measures such as hubness and betweenness centrality. This allows MetaCCI to identify differentially regulated genes that reflect condition-dependent regulatory changes across cell populations. Furthermore, unlike existing methods that primarily evaluate ligand–receptor pairs or ligand activity, MetaCCI infers cell–cell communication through an eigen-cell co-expression network constructed from these differentially regulated genes. By integrating regulatory topology with co-expression structure, MetaCCI captures network-level communication patterns and functional interaction modules among cell populations. Taken together, MetaCCI provides a systems-level framework for CCI analysis that focuses on regulatory network dynamics rather than solely ligand-driven signaling prediction, thereby offering a complementary perspective to existing approaches such as NicheNet and LIANA+.

Monte Carlo simulations demonstrated the performance of the proposed MetaCCI strategy. The simulation results indicated that MetaCCI showed consistently superior performance in CCI inference compared with existing methods. We applied MetaCCI to uncover the cell–cell communication characteristics of Myelodysplastic Syndromes (MDS). Our results identified distinct differences in interaction patterns between MDS and normal cell populations. Specifically, the loss of CCIs between “Dendritic cells and Hematopoietic precursor cells,” and between “Dendritic cells and Hematopoietic multipotent progenitor cells” has emerged as a characteristic feature of MDS. Furthermore, FABP5, CD63, and HMGB1 were identified as MDS-specific markers, whereas GSTP1, CCR7, HLA-E, and CALM2 were identified as characteristic markers of normal cells. These findings suggest key factors in the pathogenesis of MDS and potential biomarkers for disease monitoring. Our results suggest that the loss of CCIs involving dendritic cells, hematopoietic precursor cells, and multipotent progenitor cells may be a central element in MDS pathogenesis. Moreover, the delineated functional pathways and candidate markers specific to MDS or normal states provide essential knowledge for understanding the complex mechanisms underlying disease.

## Methods

We extended previous approaches for CCI inference from single gene expression level-based analysis to a gene regulatory network framework. To extract the subject of CCI inference analysis, we combined the information on ligand-receptor pairs and gene behaviors within the gene network. We then performed CCI inference using the eigen cell co-expression network in line with the data-driven approach, DD-CC-II (Park *et al.* 2025).

### Screening cell type-specific activating genes

To identify the subject of CCI inference, we screened cell type-specific activating genes based on their behaviors within the gene regulatory network.

#### Gene network estimation

For genes in the ligand-receptor database, we estimated the gene regulatory networks for the query (e.g. cancer cells) and control (e.g. normal cells) groups based on their expression levels. Suppose $X_{g}={\left(x_{1g},\ldots ,x_{n_{g}}\right)}^{T}\in R^{n_{g}\times p}$ represents the gene expression levels for the query (g=Q) and control (g=C) groups. In this study, we considered a directed gene regulatory network, and the following linear regression model was used to describe the directed molecular interplay between genes:


$$x_{i\ell g}=\sum_{j\neq \ell }^{p}x_{\mathrm{ijg}}\beta_{j\ell g}+\epsilon_{ig},\;\ell =1,\ldots ,p.$$

where $x_{i\ell g}$ is the expression level of the $\ell^{th}$ gene in the $i^{th}$ cell of group *g*. Directed gene network estimation, including both edge-weight estimation and edge selection, was performed using the following lasso approach (Tibshirani 1996):


$${\hat{\beta }}_{\ell g}=\underset{\beta_{\ell g}}{\text{arg}\;\text{min}}\{\frac{1}{2}\sum_{i=1}^{n}{\left(x_{i\ell g}-\sum_{j\neq \ell }^{p}\beta_{\ell jg}x_{\mathrm{ijg}}\right)}^{2}+\lambda \sum_{j\neq \ell }^{p}|\beta_{\ell jg}|\},$$

where λ>0 is a hyperparameter that controls the degree of shrinkage in $\beta_{\ell g}$ and is selected by minimizing the Bayesian Information Criterion (Zou *et al.* 2007). The estimated regression coefficient ${\hat{\beta }}_{\ell }$ indicates the strength of the effect of regulator genes $x_{jg}$ (j≠ℓ) on the $\ell^{th}$ target gene $x_{\ell g}$.

#### Gene activity measures

We measured gene activities based on comprehensive information about gene behaviors within a gene network, namely expression levels, hubness, and betweenness centrality.

1. Regulatory effect of the $j^{th}$ gene to its target genes
  (1) $$R_{jQ}={\bar{x}}_{jQ}\sum_{\ell \neq j}^{p}|{\hat{\beta }}_{j\ell Q}|\;\text{and}\;R_{jC}={\bar{x}}_{jC}\sum_{\ell \neq j}^{p}|{\hat{\beta }}_{j\ell C}|,$$
  where ${\bar{x}}_{jQ}$ and ${\bar{x}}_{jC}$ are the average expression levels of $j^{th}$ gene in groups *Q* and *C*, respectively. The regulatory effect describes gene activity based not only on expression levels but also on the association strength between genes. A large value of $R_{jQ}$ ($R_{jC}$) indicates that the $j^{th}$ gene exerts a substantial influence on the gene regulatory network of group *Q* (*C*).
2. Hubness
  $$H_{jQ}=\frac{\left|N_{jQ}\right|}{\left|N_{jQ}\cup N_{jC}\right|}\;\text{and}\;H_{jC}=\frac{\left|N_{jC}\right|}{\left|N_{jQ}\cup N_{jC}\right|},$$
  where $N_{jQ}$ and $N_{jC}$ are the sets of nodes (i.e. genes) that are directly linked to the $j^{th}$ gene in the networks for query and control groups, respectively. Hub genes, owing to their extensive connectivity within the gene regulatory network, can cause substantial perturbations in the entire network even with slight variations in their activity. Thus, hubness serves as an indicator of a gene’s influence and activity within the network.
3. Betweeness centrality
  (2) $$B_{jQ}=\frac{1}{\left(|V_{Q}|-1)(|V_{Q}|-2\right)}\sum_{t\neq j\neq u}\frac{a_{tu}{\left(j\right)}_{Q}}{a_{\mathrm{tuQ}}},$$
   
  $$B_{jC}=\frac{1}{\left(|V_{C}|-1)(|V_{C}|-2\right)}\sum_{t\neq j\neq u}\frac{a_{tu}{\left(j\right)}_{C}}{a_{\mathrm{tuC}}},$$
  where $0\leq B_{jQ},B_{jC}\leq 1$, $\left|V_{Q}\right|$ and $\left|V_{C}\right|$ are number of nodes (genes) in the networks for query and control groups, respectively, $a_{\mathrm{tuQ}}$ is the total number of shortest paths from the $t^{th}$ to $u^{th}$ genes and $a_{tu}{\left(j\right)}_{Q}$ is the number of these paths that pass through the $j^{th}$ gene (not where *j* is an end point). Betweenness centrality measures the influence of a gene over the flow of information between genes and thus quantifies its importance in mediating information flow within the gene regulatory network.
4. Activities of the $j^{th}$ gene in a network
  Finally, we defined gene activities based on the various activity measures within the network as follow:
  $$A_{jQ}=R_{jQ}\frac{\left(H_{jQ}+B_{jQ}\right)}{2}\;\text{and}\;A_{jC}=R_{jC}\frac{\left(H_{jC}+B_{jC}\right)}{2}.$$
  The activity scores $A_{jQ}$ and $A_{jC}$ can be interpreted as weighted regulatory effects modulated by hubness and betweenness centrality.

#### Screening differentially regulated genes between cell types

1. Difference in gene activities We propose a novel statistic that captures the distinct behaviors of genes across cell types as follow:
  (3) $$\Delta_{j}=(1-\frac{\left|N_{jQ}\cap N_{jC}\right|}{\left|N_{jQ}\cup N_{jC}\right|})|A_{jQ}-A_{jC}|,$$
  where the first term in (3) is the Jaccard distance with range [0,1] which measures the dissimilarity of the edge structure of the $j^{th}$ gene between two networks of groups *Q* and *C* (Li *et al.* 2014). The proposed statistic captures differences in gene activity by assigning weights according to structural dissimilarity between edges.
2. Access the significance of difference in gene activities to access the significance of differences in gene behaviors, we used a permutation framework and computed permutation p.value for the statistic $\Delta_{j}$.
  - Permute cells in groups and estimate two permuted networks.
  - For each pair of permuted gene networks, we computed activity measures $A_{jQ}^{\omega }$ and $A_{jC}^{\omega }$, and corresponding difference of gene activity $\Delta_{j}^{\omega }$ for ω=1,…,Ω.
  - Compute permutation p.value
    (4) $$p.\text{value}=\frac{\sum_{\omega =1}^{\Omega }I(\Delta_{j}^{\omega }\geq \Delta_{j})}{\Omega },$$

where I(·) is an indicator function.

We then selected the differentially regulated genes with a significance level α (i.e. *P*-value ≤α). Let $V_{Q}^{*}$ denote the set of differentially regulated genes in the gene network of the query group, where $|V_{Q}^{*}|=p^{*}$. In our methodology, CCIs were inferred by focusing on genes exhibiting differential regulation (i.e. the genes in $V_{Q}^{*}$).

### Cell–cell interaction inference

To infer CCIs between groups of cells, we employed the DD-CC-II which infers interactions between cell groups based on the co-expression network of eigen cells (Park *et al.* 2025).

#### Co-expression networks of eigen cells between groups

Let $X_{Q}^{*}={\left(x_{1Q}^{*},\ldots ,x_{n_{Q}}^{*}\right)}^{T}\in R^{n_{Q}\times p^{*}}$ denote the expression levels of the differentially regulated genes in $V_{Q}^{*}$.

The eigen cells of $X_{Q}^{*}$ are estimated using singular value decomposition (SVD) (Alter *et al.* 2000, Zou *et al.* 2006).


(5) $$X_{Q}^{*}=W_{Q}S_{Q}E_{Q}^{T},$$

where $S_{Q}=\text{diag}(s_{1Q},\ldots ,s_{R_{Q}Q})$ is a diagonal matrix with non-negative singular values satisfying $s_{1Q}\geq \cdots \geq s_{R_{Q}Q}$ and $W_{Q}=[w_{1Q},\ldots ,w_{R_{Q}Q}]\in R^{n_{Q}\times R_{Q}}$, and $E_{Q}=[e_{1Q},\ldots ,e_{R_{Q}Q}]\in R^{p^{*}\times R_{Q}}$ are orthogonal matrices composed of the left and right singular vectors, respectively. For the differentially regulated genes in $V_{Q}^{*}$, we also estimated eigen eigencells of the target group *T* (i.e. $E_{T}$) based on the expression levels of the target group $X_{T}^{*}\in R^{n_{T}\times p^{*}}$.

In the context of SVD, $W_{Q}$ encodes the projections of the $R_{Q}$ eigengenes within the $n_{Q}$-cell space, while $E_{Q}$ captures the contributions of $p^{*}$ genes in the $R_{Q}$-dimensional eigencell space (Alter *et al.* 2000). We regarded the eigen cells $E_{Q}$ and $E_{T}$ as the functional modules of the query and target groups of cells, respectively, and inferred CCIs by constructing a correlation network between $E_{Q}$ and $E_{T}$.

Cell–cell interaction inference was achieved through an eigen cell–based correlation network in accordance with the DD-CC-II methodology (Park *et al.* 2025). The Pearson correlation coefficient was used to quantify the association between the $i^{th}$ eigencell of the query group, $e_{iQ}$, and the $j^{th}$ eigencell of the target group, $e_{jT}$ (Rodgers and Nicewander 1988):


(6) $$\gamma_{ij}^{QT}=\frac{\sum_{k=1}^{p^{*}}\left(e_{\mathrm{kiQ}}-{\bar{e}}_{iQ}\right)(e_{\mathrm{kjT}}-{\bar{e}}_{jT})}{\sum_{k=1}^{p^{*}}{\left(e_{\mathrm{kiQ}}-{\bar{e}}_{iT}\right)}^{2}\sum_{k=1}^{p^{*}}{\left(e_{\mathrm{kjT}}-{\bar{e}}_{jT}\right)}^{2}},$$

where $e_{\mathrm{kiQ}}$ ($e_{\mathrm{kjT}}$) represents the expression value of the $k^{th}$ gene in the $i^{th}$ ($j^{th}$) eigen-cell of the query (target) group, and ${\bar{e}}_{iQ}$ and ${\bar{e}}_{jT}$ denote the corresponding average expression levels over the $p^{*}$ genes within those eigen-cells.

The eigen-cell correlation network between groups *Q* and *T* was constructed by including only significant eigen-cell pairs whose Pearson correlation coefficients yielded *P* values less than the significance level α.

#### Assess significance of CCIs

We quantified the degree of association between cell groups by evaluating whether the number of significant eigen-cell correlations exceeded expectations under a reference (null) distribution (i.e. overrepresentation analysis). Let *Z* denote the number of significant eigen-cell pairs between the query and target groups, modeled as a hypergeometric distribution:


(7) Z∼Hypergeom(N,K,n),

where *N* (or *K*) denotes the total number of possible eigen-cell pairs between query group *Q* and all cell groups, and *n* represents the total number of possible eigen-cell pairs between the query (*Q*) and target (*T*) groups. To evaluate the significance of the overrepresented eigen-cell pairs connecting the query and target groups, we employed a hypergeometric test based on the following *P*-value:


(8) $$p.\text{value}=1-\sum_{i=0}^{z-1}\frac{\left(\begin{array}{c} K\\ i \end{array}\right)\left(\begin{array}{c} N-K\\ n-i \end{array}\right)}{\left(\begin{array}{c} N\\ n \end{array}\right)},$$

where *z* denotes the observed outcome associated with the realization of *Z* and $\left(\begin{array}{c} a\\ b \end{array}\right)$ is a binomial coefficient. Following Benjamini–Hochberg correction for multiple testing, statistical significance for the association between cell groups *Q* and *T* was declared when the FDR q. value was less than or equal to the significance level α.

## Results

### Monte Carlo simulation

Monte Carlo simulations were performed to demonstrate the performance of the proposed strategy.

We used a single cell RNA sequencing (scRNA-seq) data obtained from publicly available CZ CELLxGENE database, namely, “Dissecting novel myeloid-derived cell states through single-cell RNA-Seq and its impact on clinical outcome across tumor types” (Guimaraes *et al.* 2024). The dataset was integrated from 13 single-cell studies encompassing cells derived from eight tumor types and normal tissues.

We considered eight cell types comprising more than 10 000 cells, namely Fibroblasts, epithelial cells, T cells, mononuclear phagocytes, endothelial cells, B cells, neutrophils and malignant cells, were used to infer CCIs for each cell type. Table 1 lists these cell types and their numbers. We also used a ligand-receptor pair database sourced from CellPhoneDB (Efremova *et al.* 2020).

**Table 1:** Cell types and number of cells.

Cell types	Acronym	♯ Cells
Fibroblast	FIBRO	38 893
Epithelial cell	EPITH	49 384
T cell	TCELL	118 496
Mast cell		4426
Mononuclear phagocyte	MONON	44 467
Endothelial cell	ENDOTH	25 261
B cell	BCELL	21 041
Megakaryocyte		185
Neutrophil	NEUTR	12 033
Plasmacytoid dendritic cell		1209
Malignant cell	MALIG	30 591

For the fibroblast cell type, we defined two cell groups (T1 and T2) based on 300 randomly selected cells from the 38 893 fibroblasts cells. We then extracted the expression levels of ligand and receptor genes in these 300 cells. For the control group, we randomly selected one cell type from the other seven cell types, and 300 cells were randomly selected from the selected cell type. We considered the interaction between T1 and T2 as a true positive of CCIs inference. To evaluate the true-negative scenario, we randomly selected 300 cells from cell populations other than fibroblasts, designated (F1 and F2). We considered T1 as a query group of cells and inferred CCIs of T1 with T2 (true positives) and with F1 and F2 (true negatives). The gene networks of T1 and the control groups were estimated based on the expression levels of ligand and receptor genes in each of the 300 selected cells. We then screened the differentially regulated ligand and receptor genes (i.e. $V_{T1}^{*}$) by comparing them with the control group network at a significance level of α=0.05. The eigen cell of the groups were estimated from the expression levels of the differentially regulated genes in $V_{T1}^{*}$ for the cells of each group (i.e. T1, T2, F1 and F2). The interactions between T1 and the other groups (i.e. T2, F1, and F2) were inferred using eigen cell correlation networks with hypergeometric tests. Analogous analytical approaches were used to investigate CCIs in other cell types. We emphasize that the Monte Carlo simulation was constructed in a query-centric manner. In this framework, differentially regulated genes were identified only from the query group (e.g. T1), and these genes define the regulatory signature of the query. The eigencells of all groups (T1, T2, F1, and F2) were then computed using this shared gene set. This ensures that eigencell vectors are defined in an identical feature space, allowing meaningful computation of correlation coefficients.

In the benchmark study, we evaluated our method by comparing it with representative approaches that reflect distinct analytical paradigms for inferring cell–cell interactions (CCIs). Specifically, iTALK identifies interactions based on differentially expressed ligands and receptors. CellChat models communication probability using ligand–receptor expression and signaling pathway information. REMI estimates interaction likelihood based on the correlation between ligand and receptor expression. SingleCellSignalR constructs signaling networks by integrating ligand–receptor relationships with cell-type-specific signaling information. In addition, NicheNet and LIANA+ were included, as they incorporate gene regulatory network information into CCI analysis. The strength of association between the cell groups (i.e. edge weight) was computed using existing methods. In SingleCellSignalR, the edge weight is calculated as the sum of ligand-receptor scores within each cell group (Cabello-Aguilar *et al.* 2020). The edge weight in CellChat corresponds to the total probability of ligand–receptor–mediated communication between cell groups (Jin *et al.* 2025). Edge weights in iTALK are assigned according to the average expression of ligand genes in the source cell population, using the “*cell_from_mean_exprs*” function available in the *iTALK* R package (Tyler *et al.* 2019). In REMI, the association strength between cell groups was measured by the number of ligand–receptor interactions identified across cell types (Yu *et al.* 2022). The edge weights for LIANA+ were calculated as the arithmetic mean of ligand–receptor interaction scores provided by the LIANA+ package, specifically using the “lr_means” values derived from For NicheNet, the edge weights were defined as the total number of prioritized ligands with a Pearson correlation coefficient exceeding a given threshold (e.g. 0.05), representing the cumulative activity of sender-derived ligands in predicting downstream gene expression changes in the receiver cell group. We defined the association strength of MetaCCI as –log(FDR-q. value). The simulation was performed for 50 iterations.

Figure 2 shows the association strengths (i.e. edge weights) between the cell groups across 50 iterations. Figure 2 demonstrates that our strategy effectively captures cell–cell interactions, with MetaCCI producing larger edge weights for true interactions (i.e. T1–T2) than for non-interacting pairs scenarios (i.e. T1–F1, T1–F2, T2–F1, T2–F2). Although CellChat, iTALK and LIANA+ performed well for specific cell populations, their accuracy declined when applied to other cell types.

**Figure 1:**
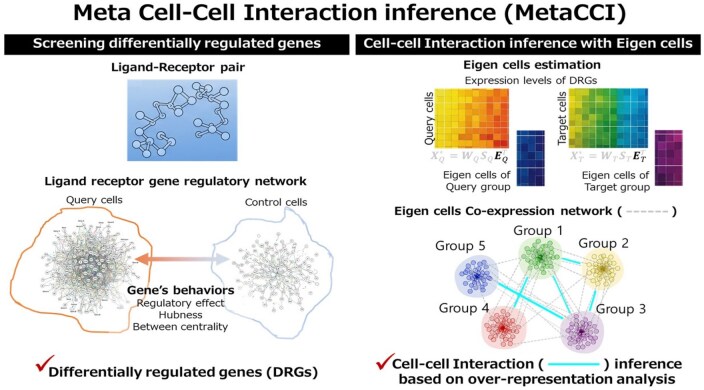
Overview of the meta cell–cell interaction inference (MetaCCI).

**Figure 2:**
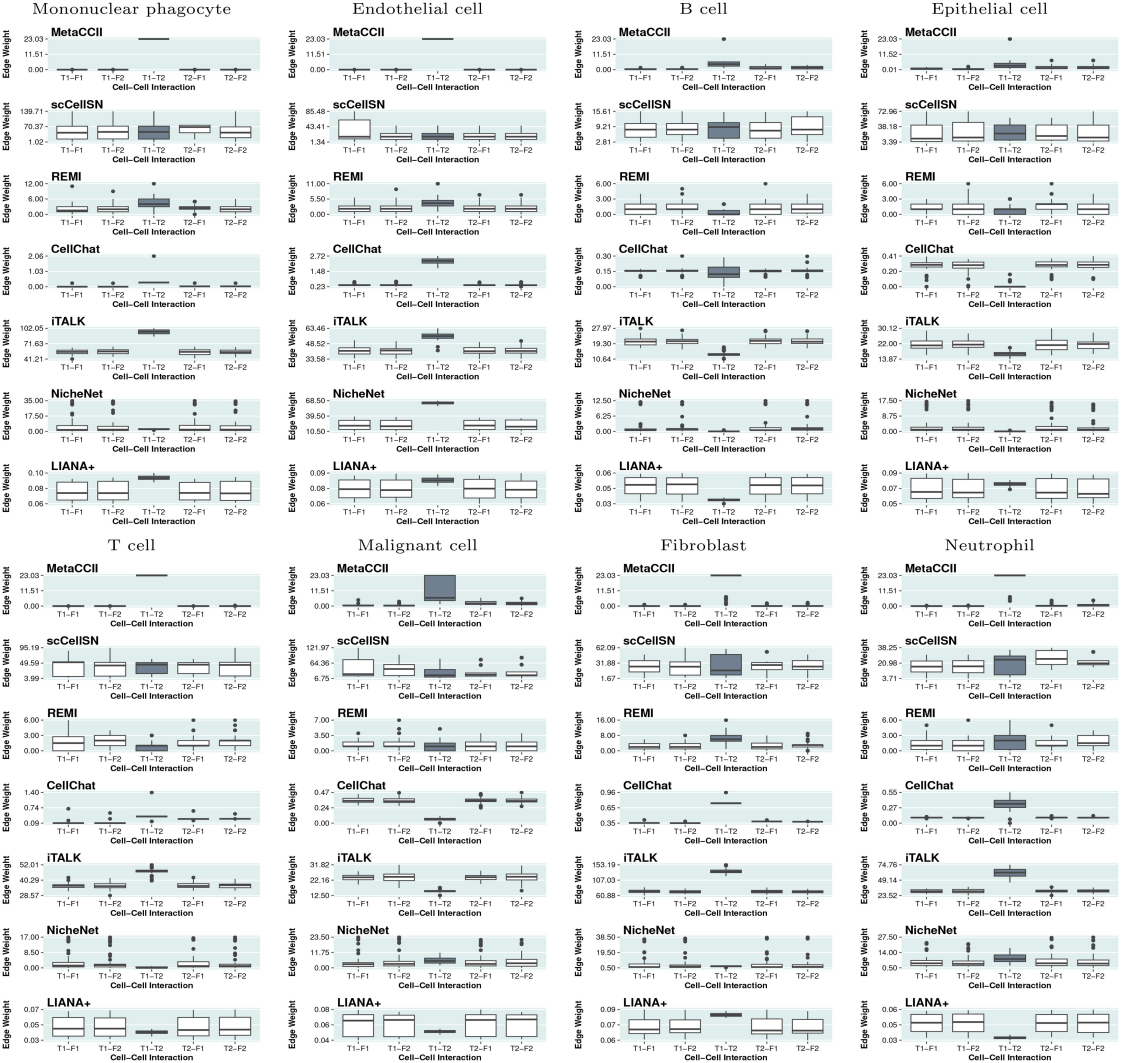
Association strength (edge weights) between cell groups is presented for each method, where T1 and T2 represent truly associated groups (ground truth), while F1 and F2 represent non-interacting groups. Each panel includes explicit numerical scales on the y-axis to facilitate quantitative comparison of inference performance. Boxplots highlighted in gray indicate the true positive interactions identified by each method. A method is considered accurate if the gray boxplot displays a higher edge weight compared to the background noise of non-interacting groups.

**Figure 3:**
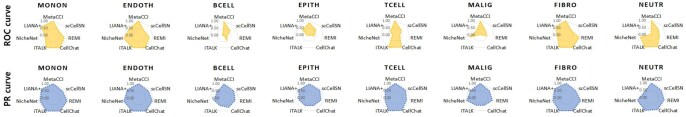
AUC values of ROC and PR curves for cell–cell interaction prediction.

We also evaluated the strategies using the area under the curve (AUC) of the receiver operating characteristic (ROC) and precision–recall (PR) curves, which are more appropriate metrics for imbalanced datasets. Figure 3 shows the AUC values of the ROC and PR curves for CCI prediction. As shown in Fig. 3, MetaCCI, iTALK, CellChat, and LIANA+ performed effectively, with MetaCCI consistently showing superior performance. In contrast, iTALK and CellChat performed poorly in detecting CCIs involving B cells, epithelial cells, and malignant cell types. In particular, the performance of LIANA+ decreased across several cell types.

We then selected the optimal threshold for the strength of association that maximized the distance to the identity (diagonal) line, based on the following optimality criterion: Youden’s J statistic (Youden 1950):


J=sensitivity+specificity−1.

The optimal threshold was determined as the value that maximized Youden’s J statistic. Using the selected threshold, we filtered the edges between cells, eliminating those with edge weights fell below the threshold. Table 2 presents the evaluation results based on various metrics for positive prediction of CCIs: accuracy, precision, recall, F1 score, true positive rate (TPR) and true negative rate (TNR).

**Table 2:** Evaluation results of cell–cell interaction inference. Table comparing the performance of multiple cell-cell interaction inference methods across different cell types using accuracy, TPR, TNR, precision, recall, and F1 score. MetaCCI generally demonstrates competitive or superior performance compared with existing methods across evaluated cell populations.^a^

Method	Cells	Accuracy	TPR	TNR	Precision	Recall	F1 score	Cells	Accuracy	TPR	TNR	Precision	Recall	F1 score
MetaCCI	MONON	**1.00**	1.00	1.00	1.00	1.00	1.00	TCELL	**1.00**	1.00	0.00	1.00	1.00	1.00
scCellSN		0.52	0.18	0.86	0.56	0.18	0.27		0.56	0.78	0.22	0.54	0.78	0.64
REMI		0.74	0.68	0.81	0.78	0.68	0.73		0.49	0.00	1.00	0.00	0.00	0.00
CellChat		**1.00**	1.00	1.00	1.00	1.00	1.00		0.98	0.98	0.02	0.98	0.98	0.98
iTALK		**1.00**	1.00	1.00	1.00	1.00	1.00		0.98	1.00	0.00	0.96	1.00	0.98
NicheNet		0.73	0.98	0.48	0.65	0.98	0.78		0.49	0.00	1.00	0.00	0.00	0.00
LIANA+		0.90	0.96	0.84	0.85	0.96	0.90		0.65	0.98	0.02	0.59	0.98	0.73
MetaCCI	ENDOTH	**1.00**	1.00	1.00	1.00	1.00	1.00	MALIG	**0.87**	0.84	0.16	0.89	0.84	0.87
scCellSN		0.51	0.60	0.42	0.51	0.60	0.55		0.53	0.24	0.76	0.56	0.24	0.34
REMI		0.71	0.76	0.66	0.69	0.76	0.72		0.50	0.02	0.98	0.57	0.02	0.04
CellChat		**1.00**	1.00	1.00	1.00	1.00	1.00		0.50	0.00	1.00	0.00	0.00	0.00
iTALK		0.98	0.96	1.00	1.00	0.96	0.98		0.50	0.00	1.00	0.00	0.00	0.00
NicheNet		**1.00**	1.00	1.00	1.00	1.00	1.00		0.69	0.90	0.10	0.63	0.90	0.74
LIANA+		0.80	1.00	0.60	0.71	1.00	0.83		0.65	1.00	0.00	0.59	1.00	0.74
MetaCCI	BCELL	**0.86**	0.96	0.76	0.80	0.96	0.87	FIBRO	0.99	1.00	0.00	0.97	1.00	0.99
scCellSN		0.60	0.50	0.70	0.62	0.50	0.55		0.62	0.30	0.70	0.81	0.30	0.44
REMI		0.50	0.00	1.00	0.00	0.00	0.00		0.85	0.84	0.16	0.85	0.84	0.85
CellChat		0.67	0.38	0.96	0.90	0.38	0.54		**1.00**	1.00	0.00	1.00	1.00	1.00
iTALK		0.50	0.00	1.00	0.00	0.00	0.00		**1.00**	1.00	0.00	1.00	1.00	1.00
NicheNet		0.50	0.00	0.99	0.00	0.00	0.00		0.70	0.88	0.12	0.65	0.88	0.75
LIANA+		0.50	0.00	1.00	0.00	0.00	0.00		0.90	1.00	0.00	0.83	1.00	0.90
MetaCCI	EPITH	**0.78**	0.78	0.77	0.77	0.78	0.78	NEUTR	0.99	1.00	0.00	0.98	1.00	0.99
scCellSN		0.60	0.84	0.35	0.56	0.84	0.67		0.67	0.60	0.40	0.70	0.60	0.65
REMI		0.50	0.00	0.99	0.00	0.00	0.00		0.57	0.52	0.48	0.57	0.52	0.54
CellChat		0.50	0.00	1.00	0.00	0.00	0.00		0.97	0.94	0.06	1.00	0.94	0.97
iTALK		0.50	0.00	1.00	0.00	0.00	0.00		**1.00**	1.00	0.00	1.00	1.00	1.00
NicheNet		0.50	0.00	1.00	0.00	0.00	0.00		0.73	0.82	0.18	0.69	0.82	0.75
LIANA+		**0.78**	1.00	0.56	0.69	1.00	0.82		0.50	0.00	1.00	0.00	0.00	0.00

a Bold values indicate the best-performing values among the compared methods.

Similar to the results from edge weight estimation and AUC analyses, MetaCCI, iTALK, and CellChat achieved reliable accuracy. Most approaches did not provide effective results when applied to epithelial-cell-type CCIs. The limited ability of certain methods (e.g. REMI) to accurately detect true CCIs appears to be a major factor underlying their poor performance for specific cell types. In summary, the proposed MetaCCI demonstrated effective performance in CCI inference, particularly in association strength estimation, AUC of ROC and PR curves, and multiple metrics for positive prediction.

To further assess the effect of subsampling size on CCI inference, we conducted a sensitivity analysis by comparing the inference accuracy and computational running time using different numbers of sampled cells (100, 300, and 1000). The results are summarized in Table 3. When 100 cells were used, the accuracy of CCI inference decreased for several cell types, suggesting that too small a sample size may lead to unstable interaction inference. In contrast, using 300 cells resulted in consistently high accuracy across most cell types. Increasing the number of cells to 1000 further improved the accuracy in some cases; however, the improvement was relatively small compared to the substantial increase in computational running time. In particular, the computational cost increased markedly as the number of sampled cells increased, while the decrease in inference accuracy when using 300 cells instead of 1000 cells remained limited. These results indicate that the number of sampled cells is an important parameter for CCI inference and that using 300 cells per cell type provides a reasonable balance between maintaining high inference accuracy and computational efficiency.

**Table 3:** Accuracy of CCI inference and computational running time obtained using different numbers of sampled cells (100, 300, and 1000) across cell types.

	Accuracy			Running time		
♯ Cells	100	300	1000	100	300	1000
MONON	0.87	1.00	1.00	1597	1698	3116
ENDOTH	0.93	1.00	1.00	1600	1882	4043
BCELL	0.97	0.86	1.00	889	1908	5690
EPITH	0.81	0.78	0.98	1014	2763	7353
TCELL	0.99	1.00	1.00	1114	2054	7372
MALIG	0.87	0.87	1.00	1447	3867	10511
FIBRO	0.78	0.99	1.00	2151	4813	10175
NEUTR	1.00	0.99	1.00	1226	2671	11238

### Evaluating spatial accuracy and biological relevance

To evaluate whether MetaCCI identifies biologically meaningful CCIs in a spatially resolved context, we analyzed the Xenium v1 Human Breast FFPE dataset with a Biomarkers and Housekeeping Genes custom panel from 10× Genomics. Specifically, we used the Xenium Output Bundle (full), which includes cell-level gene expression matrices, segmentation-derived cell annotations, metadata, and spatial coordinates generated by Xenium Onboard Analysis. These data provide single-cell-resolution transcriptomic profiles and physical cell locations within FFPE human breast tissue, allowing us to directly test whether predicted interacting cell types are spatially co-localized. Cell types were defined based on spatial clustering combined with canonical marker gene expression, leveraging cell-level transcriptomic profiles obtained directly from single-cell spatial transcriptomics data. Unlike reference-based annotation approaches, this strategy relies on aggregated gene expression within spatially resolved cells, providing high-confidence, context-specific cell-type annotations that more closely approximate true tissue-resident cell identities. This reduces ambiguity associated with broad reference atlases such as the Human Primary Cell Atlas. These annotated cell populations were used as input for downstream CCI inference.

**Figure 4:**
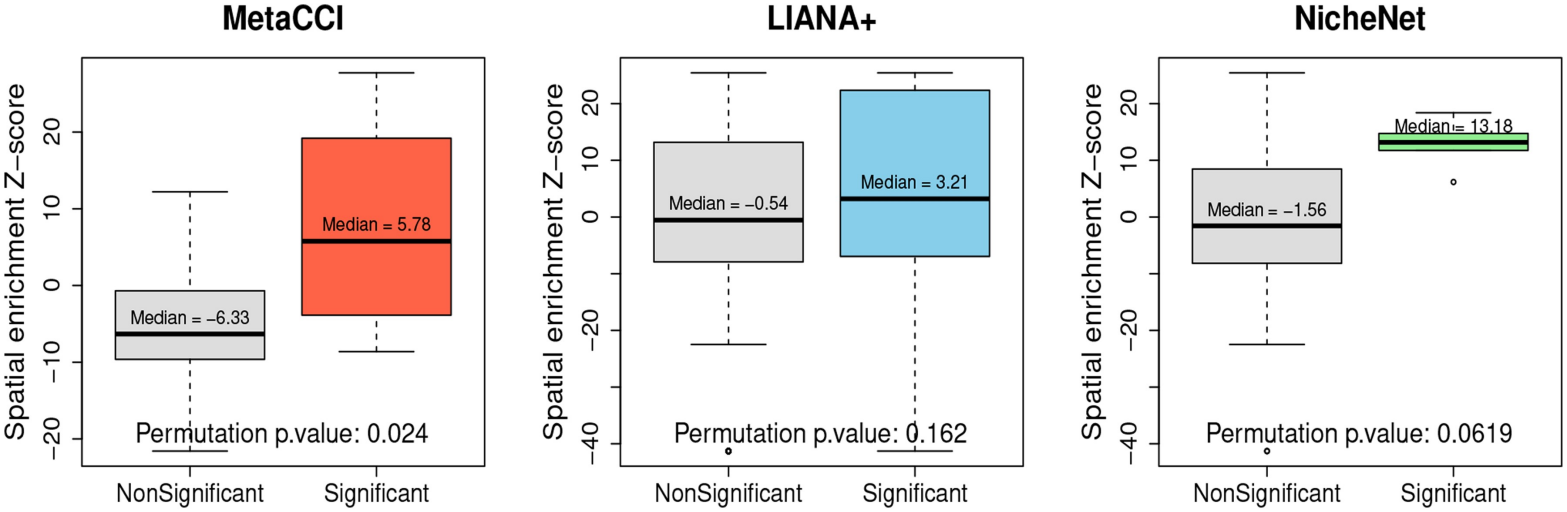
Spatial validation of predicted cell–cell interactions using MetaCCI, LIANA+, and NicheNet. Spatial enrichment *Z*-scores were computed for interactions classified as “Significant” and “Non-significant” based on a permutation-based neighborhood enrichment framework that accounts for cell-type abundance. Statistical significance between groups was assessed using permutation-based *P* values.

Benchmarking focused on network-based methods, NicheNet and LIANA+, as they integrate gene regulatory networks and are conceptually comparable to MetaCCI, whereas methods based solely on expression correlation or heuristics were excluded. For LIANA+, cell–cell interactions were inferred using the NATMI scoring method implemented in the liana_wrap function, with the Consensus ligand–receptor resource applied to log-normalized expression data. Interaction scores were defined based on the edge-specificity metric (Dimitrov *et al.* 2024). For NicheNet, ligand–receptor interactions were derived from a curated ligand–receptor network, and interaction scores were computed as the aggregated product of ligand expression in sender cell types and receptor expression in receiver cell types across all ligand–receptor pairs (Browaeys *et al.* 2020). To ensure a fair comparison, “Significant” interactions were defined using method-appropriate criteria across all methods. For MetaCCI, interactions with FDR < 0.05 were classified as “Significant,” whereas for LIANA+ and NicheNet, “Significant” interactions were defined using percentile-based thresholds applied to their respective interaction score distributions, with the top 10% of scores selected. We evaluated each method based on two criteria: (i) spatial accuracy, assessed by permutation-based spatial enrichment *Z*-scores comparing the co-localization of “Significant” and “Non-significant” interactions (Fig. 4), and (ii) biological relevance, determined by the average spatial enrichment *Z*-scores across cell-type pairs, reflecting whether predicted interactions occur between biologically meaningful and spatially proximal cell populations (Table 4).

**Table 4:** Spatial enrichment *Z*-scores for predicted cell–cell interactions between major cell-type pairs.

Cell type 1	Cell type 2	MetaCCI	LIANA+	NicheNet
ProliferativeTumor	Tumor	27.72	25.43	25.43
Perivascular	Tumor	12.22	−1.56	−1.56
Endothelial	Perivascular	10.68	18.39	18.39
Endothelial	Tumor	4.19	3.21	3.21
Endothelial	T-Lymphocytes	3.77	14.74	14.74
Endothelial	Macrophages	2.02	13.18	13.18
Perivascular	Stromal	0.88	3.48	3.48
Perivascular	T-Lymphocytes	−0.69	17.81	17.81
Perivascular	ProliferativeTumor	−1.85	0.09	0.09
Endothelial	ProliferativeTumor	−2.99	−0.54	−0.54

The *Z*-score quantifies the extent to which neighboring cell pairs occur more frequently than expected under a permutation-based null model that accounts for cell-type abundance.

To assess spatial accuracy, we employed a permutation-based neighborhood enrichment framework. Rather than using raw counts of neighboring cells, which can be biased by cell-type abundance, we quantified spatial enrichment using the following normalized *Z*-score:


$$Z_{ij}=\frac{N_{ij}^{\mathrm{obs}}-{\hat{\mu }}_{ij}^{\mathrm{perm}}}{{\hat{\sigma }}_{ij}^{\mathrm{perm}}},$$

where $N_{ij}^{\mathrm{obs}}$ denotes the observed number of neighboring cell pairs between cell types *i* and *j*, and ${\hat{\mu }}_{ij}^{\mathrm{perm}}$ and ${\hat{\sigma }}_{ij}^{\mathrm{perm}}$ represent the mean and standard deviation estimated from a permutation-based empirical null distribution. This score measures the deviation between the observed number of neighboring cell pairs and the expected number under random permutations of cell labels, thereby controlling for differences in cell-type frequency and spatial density. For each method, that is, MetaCCI, LIANA+, and NicheNet, predicted interactions were classified into “Significant” and “Non-significant” groups based on their respective inference frameworks, and their spatial enrichment *Z*-score distributions were compared. To ensure a fair head-to-head comparison, “Significant” interactions were defined in a consistent, data-driven manner using percentile-based thresholds applied to each method’s interaction score distribution. Specifically, interactions were classified as “Significant” if their scores fell within the top 10% for MetaCCI (based on spatial enrichment *Z*-scores), LIANA+ (based on edge-specificity), and NicheNet (based on aggregated ligand–receptor expression scores), while all remaining interactions were considered “Non-significant.” Statistical significance was evaluated using permutation-based *P* values.

As shown in Fig. 4, MetaCCI demonstrated a clear separation between “Significant” and “Non-significant” interactions, with significantly higher spatial enrichment for the “Significant” group (permutation p.value = 0.024). In contrast, LIANA+ and NicheNet did not show statistically significant differences between the two groups, indicating weaker spatial specificity. These results indicate that MetaCCI more effectively captures spatially co-localized interactions beyond what would be expected by random chance. The spatial accuracy metric evaluates whether interactions classified as “Significant” are more spatially co-localized than those classified as “Non-significant.” By using permutation-based normalization, this approach controls for differences in cell-type abundance and spatial density, ensuring that enrichment is not driven by highly abundant cell populations. Therefore, a higher spatial enrichment *Z*-score indicates that predicted interactions are more likely to reflect true spatially constrained cell–cell communication.

To further assess biological relevance, we aggregated predicted interactions at the cell-type level and computed the average spatial enrichment *Z*-score for each cell-type pair across all methods (MetaCCI, LIANA+, and NicheNet). This aggregated score provides a quantitative measure of whether interactions between specific cell populations are consistently observed in spatial proximity. The spatial enrichment *Z*-score quantifies the extent to which neighboring cell pairs occur more frequently than expected under a permutation-based null model that accounts for cell-type abundance. Higher *Z*-scores indicate stronger spatial co-localization and, consequently, greater biological plausibility of the inferred interactions. Table 4 summarizes the top-ranked cell-type interactions based on MetaCCI spatial enrichment scores. MetaCCI identified strongly enriched interactions between biologically plausible cell-type pairs, including proliferative tumor cells and tumor cells, endothelial cells and perivascular cells, and endothelial cells with immune populations such as macrophages and T lymphocytes. These interaction patterns are consistent with known features of the tumor microenvironment, including tumor proliferation, angiogenesis, and immune–stromal interactions (Carmeliet 2005, Hanahan and Weinberg 2011, Quail and Joyce 2013).

In contrast, LIANA+ and NicheNet exhibited inconsistent spatial enrichment patterns across cell-type pairs. LIANA+ frequently assigned elevated enrichment scores across diverse cell-type combinations, suggesting potential overprediction, whereas NicheNet showed reduced sensitivity in detecting several biologically expected interactions, indicating possible false negatives. These discrepancies highlight differences in how each method balances sensitivity and specificity in CCI inference. The biological relevance metric complements the spatial accuracy analysis by identifying cell-type interactions that are not only statistically enriched but also consistent with known biological organization. Together, these results demonstrate that MetaCCI achieves improved spatial accuracy and biological relevance by capturing interactions that are both spatially constrained and biologically meaningful, while avoiding overprediction and underdetection observed in alternative methods.

### Cell–cell communication characteristics of MDS

Myelodysplastic syndromes (MDS) are a group of clonal hematopoietic disorders characterized by defective hematopoiesis, which leads to persistent cytopenia, morphological dysplasia in bone marrow cells, and an increased risk of transformation to acute myeloid leukemia (Zhou *et al.* 2011). Understanding intercellular signaling in MDS is vital for identifying novel targets that may restore normal hematopoietic function and block disease-promoting pathways (Medyouf 2017, Tosato *et al.* 2021).

We applied our strategy, MetaCCI, to uncover the characteristics of CCIs in MDS. We used the publicly available scRNA-seq data “Human circulating hematopoietic stem and progenitor cells in aging, cytopenia and MDS (human cHSPCs—Illumina)” provided by CZ CELLxGENE database (Furer 2025). The dataset consisted of four cell types: hematologic disorder, myelodysplastic syndromes, myelodysplastic/myeloproliferative disease, and normal cells. We analyzed 62 092 MDS cells and 342 712 normal cells to characterize CCIs within the MDS group by comparing them with those in the normal cell population. Table 5 lists the types and numbers of cells in the MDS and normal populations.

**Table 5:** Cell types and number of cells for MDS and normal populations.

Cell types	MDS	Normal
Antibody secreting cell	9	73
B cell	3835	82 178
Dendritic cell	1076	965
Endothelial cell	36	260
Hematopoietic multipotent progenitor cell	32 255	109 546
Hematopoietic precursor cell	16 011	125 266
Lymphocyte	355	2888
Monocyte	2869	9157
Unknown	678	3756

Based on the Monte Carlo simulation results indicating that 300 cells provide a reasonable balance between computational efficiency and CCI inference accuracy, we used 300 randomly selected cells per cell type for the analysis. Accordingly, only cell types with at least 300 cells in both MDS and normal populations (B cells, dendritic cells, hematopoietic multipotent progenitor cells, hematopoietic precursor cells, lymphocytes, and monocytes) were included, while cells of unknown type were excluded.

**Figure 5:**
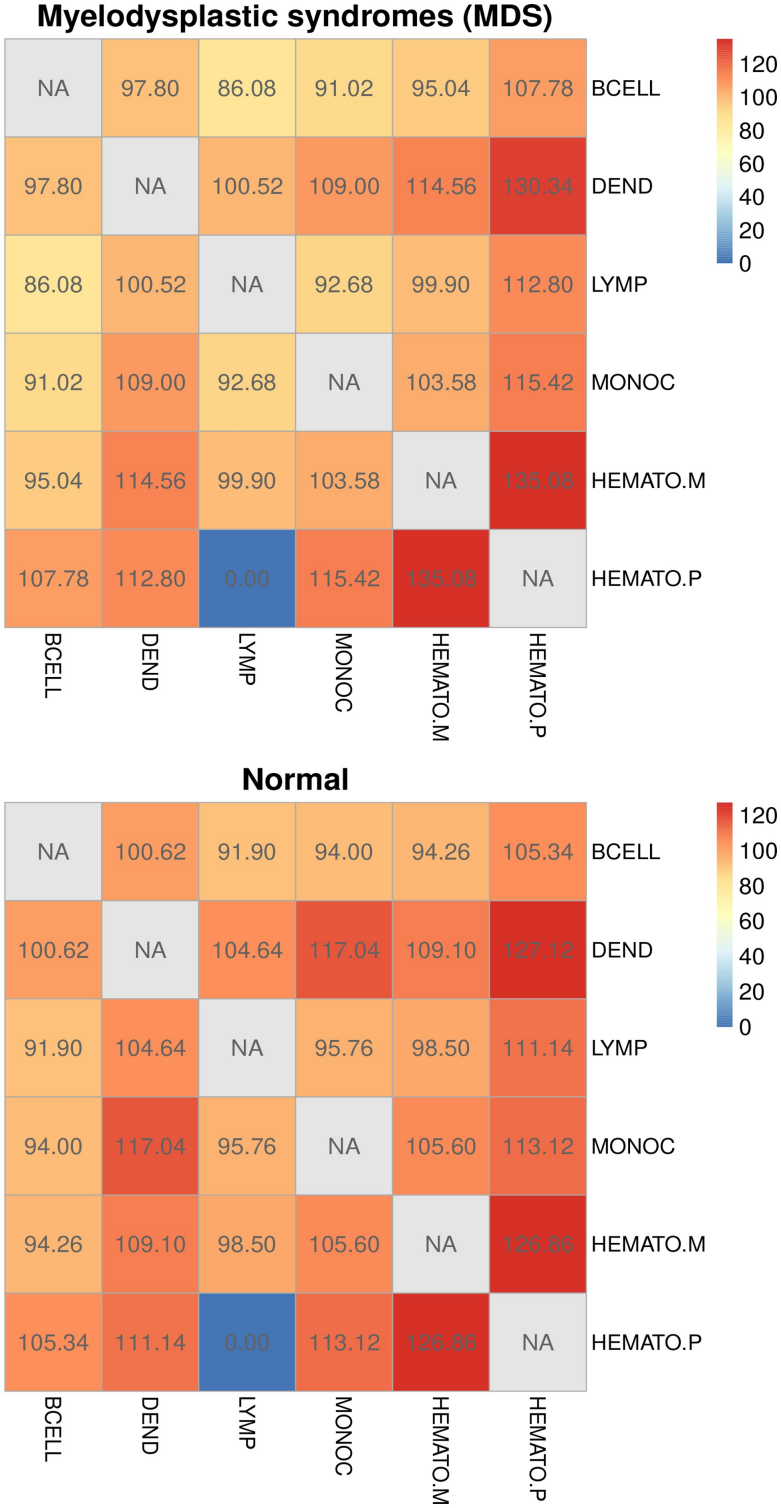
Eigen cell correlation networks in normal and MDS cell populations. Each heatmap represents the correlation strength between query and target cell populations, defined as the mean number of significantly correlated eigen cell pairs across 50 independent iterations. Higher values indicate stronger eigencell associations between cell populations. Numbers within each cell denote the average count of significant correlations, and gray cells (NA) represent intra-population diagonal comparisons.

As each cell type has a different population size and affects the estimated gene network structure, we randomly selected 300 cells from each group of cells and estimated the gene networks for six cell groups in the MDS and normal populations. Specifically, we estimated the gene networks of B cell groups using 300 randomly selected cells from the ligand-receptor pair database, where the 300 cells were chosen from 3835 and 82 178 B cells, respectively. The gene network of the control group was estimated by randomly selecting 300 cells from one of the five cell types other than B cells. By comparing the gene behaviors in the B cell networks and the control groups, we extracted differentially regulated genes within the B cell network (i.e. $V_{\mathrm{Bcell}}^{*}$) for MDS and normal population, respectively. The eigen cells of the B cells were estimated using the expression levels of the differentially regulated genes in 300 randomly selected cells (i.e. $E_{\mathrm{Bcell}}$). For genes in $V_{\mathrm{Bcell}}^{*}$, we estimated eigen eigenvalues across five additional cell types and subsequently constructed an eigen cell co-expression network encompassing all six cell groups. CCIs between B cells and the other five groups were inferred based on significant eigen cell pairings, determined via a hypergeometric test. To establish a baseline for healthy CCI, we first analyzed the eigen cell correlation network in a normal cell population using the same methodology, quantifying correlation strength as the mean number of significantly correlated eigen cell pairs over 50 iterations. Figure 5 shows the eigen cell correlation network in a normal cell population. *Conserved hematopoietic lineage connectivity*: In both normal and MDS groups, the strongest correlation was consistently observed between HEMATO.M and HEMATO.P (normal: 126.86; MDS: 135.08). This strong association reflects the close developmental relationship between hematopoietic progenitors and mature hematopoietic cells, suggesting that MetaCCI successfully captures biologically meaningful lineage connectivity (Furer *et al.* 2025). *Robust myeloid communication axis*: Strong correlations were also observed between DEND and MONOC (normal: 117.04; MDS: 109.00) and between DEND and HEMATO.P (normal: 127.12; MDS: 130.34). These interactions indicate an active signaling axis within the myeloid lineage, which is consistent with the known role of dendritic cells and monocytes in immune regulation and hematopoietic microenvironment signaling (Medyouf 2017). *Consistent lineage separation*: Notably, the correlation between LYMP and HEMATO.P was 0.00 in both normal and MDS networks. This consistent absence of correlation suggests that MetaCCI effectively distinguishes between biologically unrelated developmental lineages, which is consistent with the well-established divergence between lymphoid and myeloid differentiation pathways in hematopoiesis (Murphy *et al.* 2016). *Disease-associated network modulation*: Although the overall lineage structure is preserved, several correlation strengths differ between the normal and MDS networks, indicating potential disease-associated modulation of cell–cell communication. These results suggest that MetaCCI can capture both conserved lineage relationships and pathological alterations in CCC networks in MDS (Medyouf *et al.* 2014).

CCIs with FDR-q. values less than α=0.05 were considered significant. To ensure the statistical reproducibility of our findings and to minimize potential sampling bias, these procedures for CCI inference were conducted through 50 independent iterations, with each iteration utilizing 300 cells randomly subsampled from the respective populations. To filter out stochastic noise and identify robust communication patterns, only interactions that consistently appeared in more than 50% of the iterations (i.e. at least 25 out of 50) were defined as significant interactions between cell groups. The association strength (edge weight) was subsequently determined by averaging the results across these 50 iterations. The association strength between cell groups (i.e. edge weight) was computed as the mean of -log(FDR-q. value) over 50 iterations. For the other five cell groups, CCIs were inferred using analogous analysis steps.

**Figure 6:**
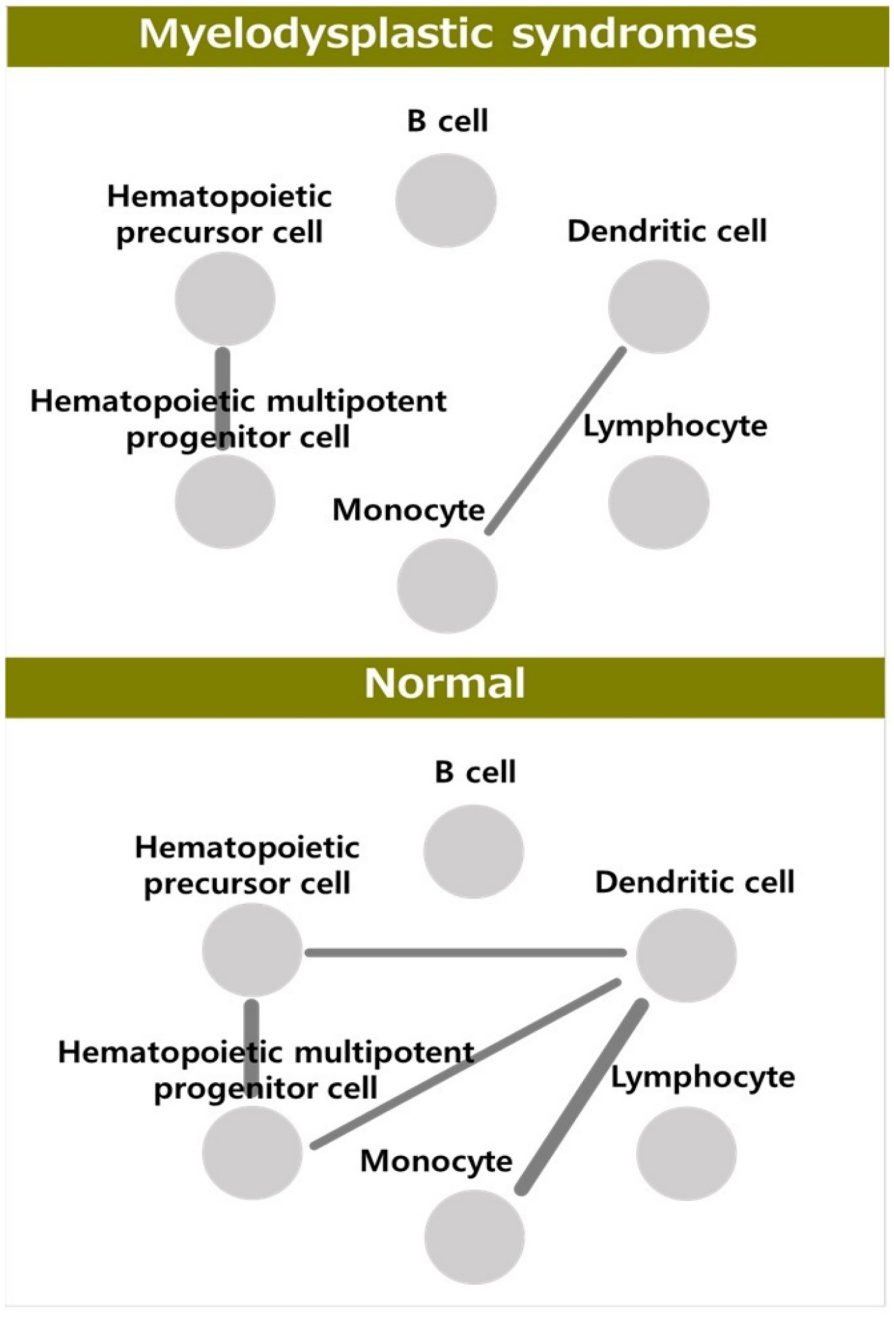
Cell–cell interaction of MDS and normal cells, where edge thickness represents the edge weight (i.e. strength of association between cell groups).

Figure 6 shows the CCIs for the cell groups of MDS and normal populations.

As shown in Fig. 6, interactions between “Dendritic cell with Monocyte” and between “Hematopoietic multipotent progenitor cells and Hematopoietic precursor cell” were observed as common CCIs in both MDS and normal populations. The loss of CCIs between “Dendritic cells and Hematopoietic precursor cell,” as well as between “Dendritic cell and Hematopoietic multipotent progenitor cells,” was identified as a distinctive feature of MDS. Additionally, the hubness of Dendritic cells emerged as a characteristic specific to normal conditions. Thus, communication between dendritic cells, hematopoietic precursors, and multipotent progenitor cells is essential for elucidating the mechanisms of MDS.

- *Dendritic cells* Dendritic cells are pivotal mediators of immune regulation, orchestrating both the initiation and modulation of immune responses (Van *et al.* 2022). Van *et al.* (2022) suggested that dysfunctional responses of dendritic cell subsets to cellular stress and DNA damage contribute to immune evasion and disease progression in MDS. It was also demonstrated that, within MDS and AML, the dendritic cells arise from malignant progenitors and exhibit numerical reduction and functional dysregulation, influencing the immune milieu and contributing to disease evolution (Panoskaltsis 2005).
- *Hematopoietic multipotent progenitor cell* Hematopoietic multipotent progenitor cells originate from hematopoietic stem cells and represent an intermediate stage in hematopoiesis. In contrast to hematopoietic stem cells, hematopoietic multipotent progenitor cells possess restricted self-renewal potential, yet maintain the capacity to generate diverse blood cell lineages (Doulatov *et al.* 2025).
- *Hematopoietic precursor cell* Autologous cytotoxic responses targeting hematopoietic progenitor cells were mediated by natural killer cells, suggesting a supplementary function of the innate immune system in the immunosurveillance of patients with MDS (Chamuleau *et al.* 2009).

**Figure 7:**
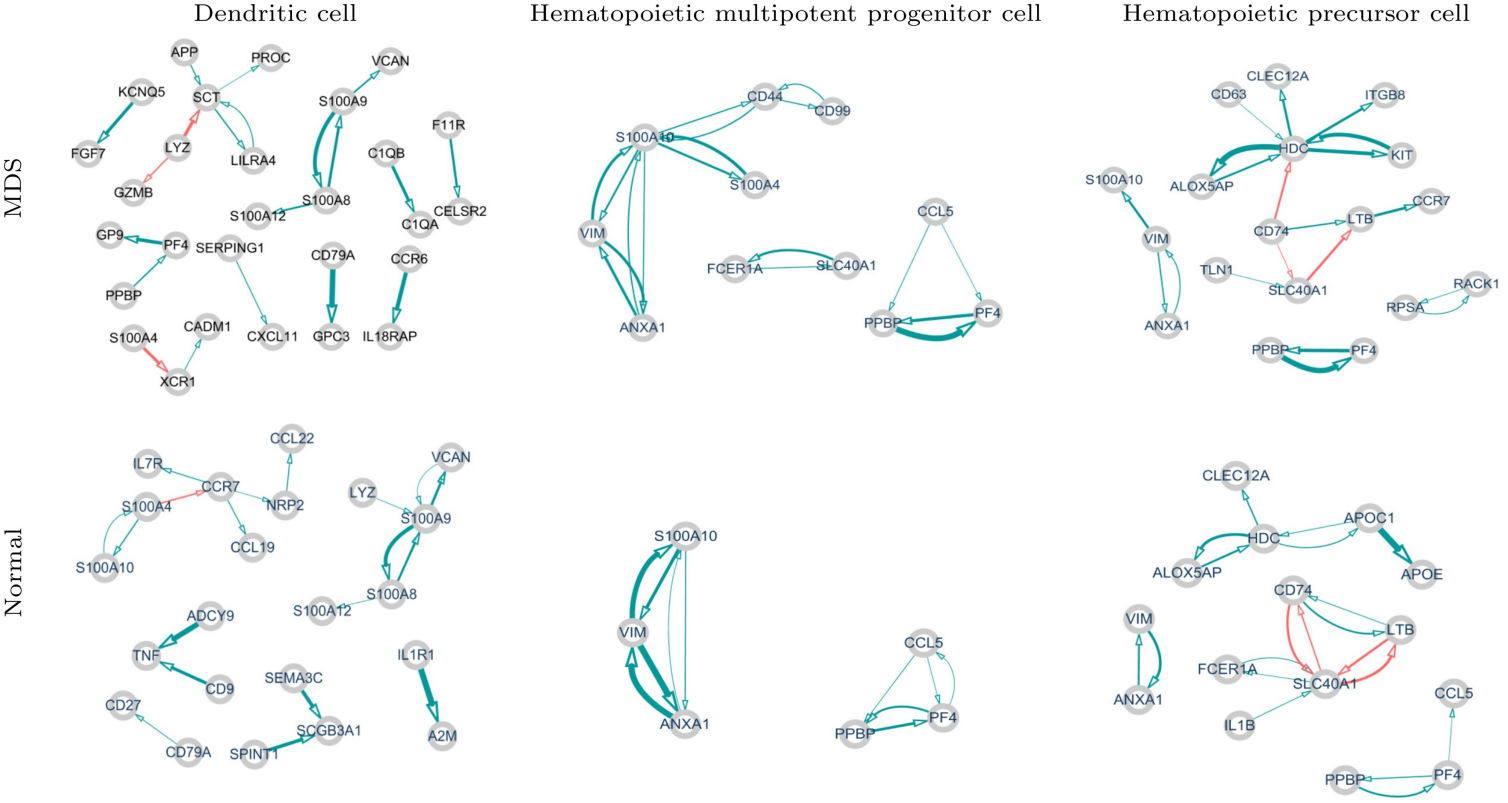
Molecular interplays among differentially regulated genes within each cell group, where arrows (⊕→⊗) denote strength of effect from ⊕ on ⊗, edge thickness reflects edge weight, and edge color represents the sign of regulation (blue and red correspond to positive and negative interactions, respectively).

To reveal the cell type-specific characteristics of crucial cell groups, the molecular interactions of differentially regulated genes were analyzed within each cell type. Figure 7 shows the gene networks of the differentially regulated genes in each cell type. From the edges estimated across 50 iterations, we visualized the top 20 edges with the largest median absolute weights to represent the complex gene network. Edge weights were defined as the median values across the 50 iterations.

As shown in Fig. 7, the molecular interplay differs substantially between MDS and normal populations. The crucial cell types in MDS display relatively higher molecular interaction activity, that is, greater edge and node counts, than their counterparts in the normal population.

**Figure 8:**
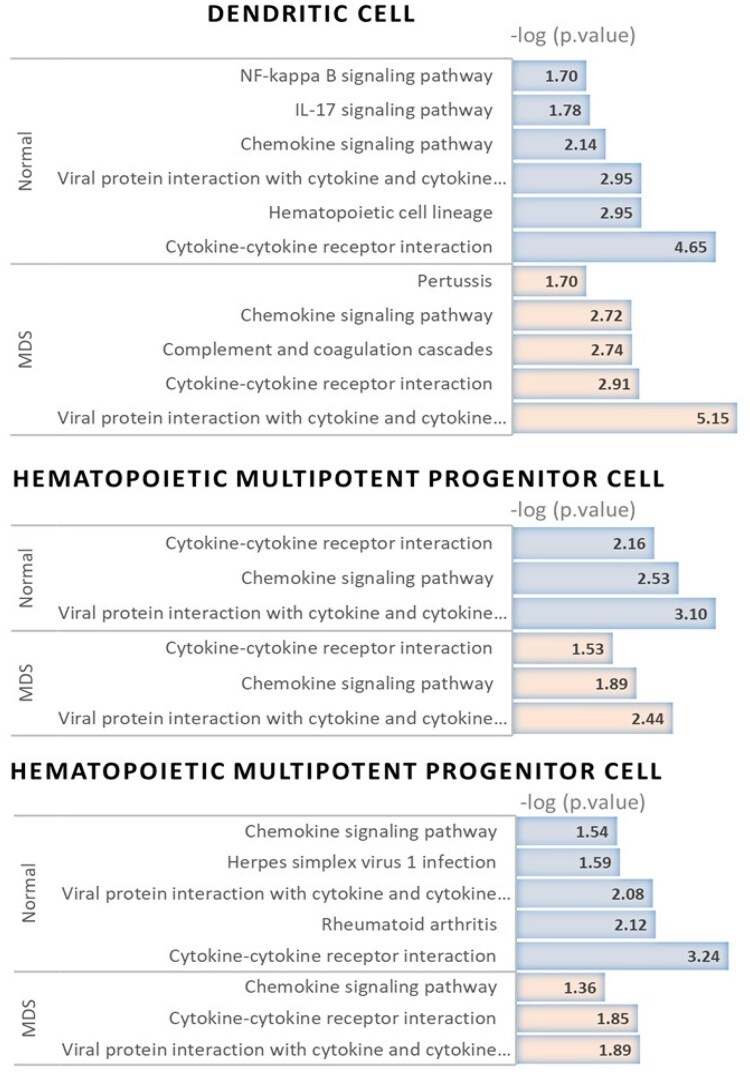
KEGG pathway analysis results for cell type specific gene networks.

To identify the biological functions involved in the gene networks of cell groups, we performed Kyoto Encyclopedia of Genes and Genomes (KEGG) pathway analysis. Figure 8 shows the significantly enriched functional pathways of the molecular interplays (*P*-value ≤ .05) that characterized the cells of groups for MDS and normal populations.

The results demonstrate that “Viral protein interaction with cytokine and cytokine receptor,” “Chemokine signaling pathway” and “Cytokine-cytokine receptor interaction” are common enriched functional pathways for most of gene networks. In the gene network of dendritic cells, “Pertussis” was identified as a functional pathway in the MDS, whereas “IL-17 signaling pathway” and “NF-kappa B signaling pathway” were identified as functional pathways in the normal populations. Functional pathway analysis revealed that the hematopoietic precursor cell network in the normal group was associated with “Rheumatoid arthritis” and “herpes simplex virus 1 infection.” Previous studies have reported that functional analysis of MDS and AML revealed that differentially expressed genes (DEGs) were significantly enriched in pathways related to Th17 cell differentiation, pertussis, and cytokine–receptor interactions, suggesting potential involvement of immune regulatory cells such as dendritic cells in the pathogenesis of MDS (Liang *et al.* 2022). Xu *et al.* (2025) demonstrated that transcriptomic analyses of MDS have identified the IL-17 signaling pathway as significantly enriched in KEGG pathway analysis, suggesting its role in inflammatory immune responses in the MDS microenvironment. In MDS, increased expression of TLR2 and TLR4 in hematopoietic stem and progenitor cells activates inflammasome signaling and enhances IL-1β production, thereby triggering the noncanonical NF-κB pathway and promoting an inflammatory microenvironment (Passos *et al.* 2025). Barreyro *et al.* (2018) demonstrated that TLR4-mediated signaling in hematopoietic stem/progenitor cells promotes myeloid-biased hematopoiesis in immune-related diseases associated with MDS, such as arteriosclerosis and rheumatoid arthritis. Previous transcriptomic network analyses in MDS revealed significant enrichment of several gene regulatory networks in the KEGG herpes simplex virus infection pathway, suggesting a potential role of antiviral and immune-related signaling in MDS pathogenesis (Wang *et al.* 2013).

We then identify crucial genes involved in CCIs between cell groups, that is, the top ten genes with the largest absolute loading values for the first eigen cell estimation, where each absolute loading value was computed as an average over 50 iterations. Table 6 shows the crucial genes for CCIs of each cell group.

**Table 6:** Markers for cell–cell interaction of cell groups, where text colors indicate MDS (*), Normal (#) -specific and common (gray) markers.

Rank	Dendritic cell		Hematopoietic M		Hematopoietic P	
	MDS	Normal	MDS	Normal	MDS	Normal
1	FABP5*	S100A10	VIM	VIM	HDC	SLC40A1
2	TIMP1	HSP90B1	CD44	ITGA4	SLC40A1	LTB
3	S100A9	GSTP1${\;}^{\#}$	S100A4	HLA-E${\;}^{\#}$	VIM	VIM
4	CD63*	CCR7${\;}^{\#}$	HLA-C	SLC40A1	S100A4	CALM2${\;}^{\#}$
5	VIM	S100A4	HLA-A	CALM2${\;}^{\#}$	CD63*	CCR7${\;}^{\#}$
6	CALR	GZMB	SELL	SELL	LTB	GSTP1${\;}^{\#}$
7	S100A8	S100A8	TFPI	AVP	FABP5*	S100A4
8	APLP2	LYZ	S100A10	S100A4	KIT	CALR
9	LTB	S100A9	HSP90B1	GSTP1${\;}^{\#}$	HMGB1*	HLA-E${\;}^{\#}$
10	HMGB1*	VCAN	FABP5*	CD44	TFRC	HDC

In line with the findings presented above, “Dendritic cells,” “Hematopoietic multipotent progenitor cells,” and “hematopoietic precursor cells” for MDS and normal populations possess distinct sets of crucial genes. The results indicated that FABP5, CD63, and HMGB1 functioned as MDS-specific markers, whereas GSTP1, CCR7, HLA-E, and CALM2 were specific to the normal population. These findings suggested that markers specific to both MDS and normal cells may serve as valuable indicators for monitoring MDS progression. The identified MDS-specific markers, CD63 and HMGB1, have strong supporting evidence for their role as biomarkers in MDS.

- CD63: CD63 expression was significantly diminished in MDS, with frequency reduced in low-risk cases and MFI decreased in high-risk cases (Liu *et al.* 2020). Sandes *et al.* (2012) systematically evaluated the quantitative expression of platelet glycoproteins (e.g. CD42b, and CD61), together with platelet activation-associated markers (PAC-1, CD62P, fibrinogen, and CD63), as well as platelet light scatter properties. They express typical MSC markers (CD29, CD44, CD73, CD105) and additionally express tetraspanins (CD81, CD82, CD63, CD53, CD9, CD37) due to their endosomal origin (Batsali *et al.* 2020).
- HMGB1 Apodaca-Chávez *et al.* (2022) showed that circulating HMGB1 is elevated in MDS and can differentiate MDS from other bone marrow failure disorders, highlighting its possible mechanistic and therapeutic relevance. HMGB1 blockade may enhance MDS cell apoptosis and modulate innate immune responses through inhibition of NF-κB signaling (Kam *et al.* 2019).

Although a direct association between FABP5 and MDS has not yet been established, key mechanistic roles of FABP5 in leukemia have been demonstrated. For example, FABP5 targeting induces apoptosis and suppresses triglyceride production in AML, while its inhibition activates retinoic acid signaling and promotes myeloid differentiation in AML cells (Liang *et al.* 2023). In addition, FABP5 has been shown to reprogram lipid metabolism and promote disease progression in cutaneous T-cell lymphoma through activation of PPARγ signaling (Wang *et al.* 2025).

Taken together, our findings suggest that loss of CCIs between “Dendritic cell” with “Hematopoietic precursor cell” and “Hematopoietic multipotent progenitor cell” may provide crucial clue to uncover progress of MDS. In addition, the functional pathways and critical markers identified in MDS and normal populations provide important insights into the mechanisms driving MDS.

#### Cross-platform validation and robust signaling of MDS markers

To evaluate the robustness of the identified biomarkers (FABP5, CD63, and HMGB1) and the comparative efficacy of MetaCCI, we performed an extensive cross-platform validation using six ordinary CCI inference strategies, that is, SingleCellSignalR, LIANA, CellChat, iTALK, REMI, and NicheNet. This multi-algorithmic approach was designed to verify whether these specific markers could be consistently recovered using existing methodologies under standardized criteria. The validation process followed a rigorous pipeline, beginning with the identification of statistically significant CCIs within each platform through permutation p.values (i.e. p.values<0.05) to ensure the reliability of the inferred interactions. Within these significant communication networks, we then extracted the top 10 most influential genes by tailoring the definition of “importance” to the specific scoring metrics of each methodology. For platforms such as CellChat, iTALK, REMI, and LIANA, importance was quantified by the communication strength, derived from the joint expression levels of ligands and receptors to represent the absolute intensity of the signaling flux. In contrast, SingleCellSignalR utilized the LRscore to evaluate the functional probability and reliability of specific ligand-receptor pairs, while NicheNet defined importance through “Ligand Activity,” a metric that reflects a ligand’s functional impact by its capacity to trigger downstream transcriptional changes in receiver cells. Finally, we conducted a cross-platform consensus analysis to verify the consistent presence of FABP5, CD63, and HMGB1 within these high-ranking interactions across all methodologies. The results of this comprehensive validation, summarized in Table 7, where Max Consensus denotes the maximum number of methods (out of six) that cross-identified a specific interaction, and High-Confidence Pathways refers to the number of communication routes consistently supported by at least three independent methods.

**Table 7:** Cross-platform validation of MDS-specific markers across six CCI algorithms.

Markers	Max	High-confidence	Validated platforms
	Consensus	**Paths (** ≥3 **)**	
CD63	4/6	17	CellChat, iTALK,
			LIANA, REMI
FABP5	3/6	6	LIANA, REMI, scCellSN
HMGB1	6/6	18	CellChat, iTALK, LIANA,
			NicheNet, REMI, scCellSN

As shown in Table 7, the identified markers are not platform-specific artifacts but are robust biological drivers of the MDS microenvironment. Notably, HMGB1 achieved a perfect consensus across all methods, while CD63 and FABP5 were validated by four and three independent algorithms, respectively. This high degree of cross-platform agreement underscores the ability of MetaCCI to mitigate individual algorithmic biases and prioritize high-confidence signaling mediators that are likely to be experimentally confirmed.

## Discussion

We introduced a novel computational strategy, MetaCCI, for cell–cell interaction inference. MetaCCI integrates established ligand–receptor pairs with quantitative insights into gene behavior within complex gene networks. By combining these complementary aspects of gene expression and gene behaviors, MetaCCI accurately identifies the most relevant elements for CCI analysis. Subsequently, CCIs are inferred using an eigen cell co-expression network. This approach enables the identification of functional modules within cell populations and facilitates the inference of interactions between these modules, offering a more comprehensive view of cell–cell communication. Thus, we could effectively infer CCIs and interpret CCIs results based on molecular interactions within cell types. Monte Carlo simulations validated the utility of the proposed strategy. Monte Carlo simulations provided evidence supporting the efficacy of our approach for CCI inference. We further applied MetaCCI to characterise the CCIs of MDS cells compared with those of normal cell populations. Our results revealed the loss of CCIs between “Dendritic cell with Hematopoietic precursor cell,” as well as between “Dendritic cell with Hematopoietic multipotent progenitor cell,” as a distinctive characteristics of MDS. In addition, our findings indicate that distinct genes and interaction patterns predominantly govern the CCIs of MDS compared with those in normal populations. Our results suggest that diminished CCIs involving dendritic cells with hematopoietic precursors and multipotent progenitor cells may be a pivotal factor in MDS development. Additionally, the functional pathways and candidate markers identified in MDS versus normal populations offer valuable information for understanding the disease’s underlying molecular mechanisms.

Recent advances in cell–cell interaction (CCI) analysis have increasingly emphasized the importance of incorporating additional biological context beyond conventional ligand–receptor expression analysis. In particular, several computational frameworks aim to integrate intracellular regulatory information or spatial context to better characterize intercellular communication (Ma *et al.* 2024, Zhang *et al.* 2025). Previous studies have also highlighted that intracellular signaling cascades and gene regulatory networks play critical roles in determining downstream cellular responses to ligand–receptor interactions (Efremova *et al.* 2020, Jin *et al.* 2025). In parallel, the emergence of spatial transcriptomics technologies has enabled spatially informed CCI inference methods. For example, SpaOTsc models signaling relationships between spatially proximal cells using optimal transport theory (Cang and Nie 2020), while Giotto integrates spatial gene expression with cellular neighborhood structures to identify spatially constrained communication networks (Dries *et al.* 2021). By incorporating spatial proximity information, these approaches improve the biological realism of inferred interactions, as cell–cell communication is often shaped by the spatial organization of cells within tissues. Together, these developments highlight the importance of integrating multiple layers of biological information for a more comprehensive understanding of intercellular communication. In this context, the network-based framework of MetaCCI provides a flexible foundation for future methodological extensions, such as incorporating spatial transcriptomic information or coupling with more detailed intracellular signaling models.

## Data Availability

The datasets analyzed in this study are publicly available from the CZ CELLxGENE database. The data can be accessed at https://cellxgene.cziscience.com/.
